# Bromelain-loaded nanocomposites decrease inflammatory and cytotoxicity effects of gliadin on Caco-2 cells and peripheral blood mononuclear cells of celiac patients

**DOI:** 10.1038/s41598-023-48460-3

**Published:** 2023-12-01

**Authors:** Masoumeh Sadat Mousavi Maleki, Ramin Ebrahimi kiasari, Seyed Javad Seyed Mousavi, Hamid Hashemi‐Moghaddam, Ali Akbar Shabani, Hamid Madanchi, Soroush Sardari

**Affiliations:** 1 Nervous System Stem Cells Research Center, Semnan University of Medical Sciences, Semnan, Iran; 2 Department of Biotechnology, School of Medicine, Semnan University of Medical Sciences, Semnan, 35131-38111 Iran; 3 Gene Therapy and Regenerative Medicine Research Center, Hope Generation Foundation, University of Social Welfare and Rehabilitation Sciences, Tehran, Iran; 4 Drug Design and Bioinformatics Unit, Medical Biotechnology Department, Biotechnology Research Center, Pasteur Institute of Iran, Tehran, 13198 Iran; 5 Department of Chemistry, Damghan Branch, Islamic Azad University, Damghan, Iran

**Keywords:** Biochemistry, Biotechnology, Molecular biology

## Abstract

Enzyme therapy can be an appropriate treatment option for celiac disease (CeD). Here, we developed Bromelain-Loaded Nanocomposites (BLNCs) to improve the stability and retention of bromelain enzyme activity. After the characterization of BLNCs, the cytotoxicity of BLNCs was determined on the Caco-2 cell line. The effect of BLNCs on gliadin degradation and the production of pro-inflammatory cytokines and anti-inflammatory molecules in peripheral blood mononuclear cells (PBMCs) obtained from celiac patients were assessed. Furthermore, the expression of CXCR3 and CCR5 genes was measured in CaCo-2 cells treated with gliadin, gliadin-digested with BLNCs, and bromelain. Our study demonstrated that the Bromelain entrapment efficiency in these nanoparticles was acceptable, and BLNCs have no toxic effect on cells. SDS-PAGE confirmed the digestion effect of bromelain released from nanocomposites. When Caco-2 cells were treated with gliadin digested by free bromelain and BLNCs, the expression of CXCR3 and CCR5 genes was significantly decreased. PBMCs of celiac patients treated with Bromelain and BLNCs decreased inflammatory cytokines (IL-1β, IL-6, TNF-α, and IFN-γ) production compared to untreated PBMCs. This treatment also increased IL-10 and CTLA-4 in PBMCs of CeD patients. According to the promising results of this study, we can hope for the therapeutic potential of BLNCs for CeD.

## Introduction

Celiac disease (CeD) is one of the most common autoimmune disorders that occur with exposure to dietary gluten in genetically predisposed individuals and affects about 1% of the population worldwide. Gluten is a protein mixture consisting of gliadin and glutenin, defined as prolamin and insoluble in both water and dilute saline solutions, and resistant to proteolytic breakdown by gastric and intestinal digestive proteases^1^. Also, gliadins are protein mixtures that these proteins classified into Sulfur-rich (α, β, and γ gliadins) and Sulfur-poor (ω gliadins) based on their amino acid sequence^2^. Glutamine-rich sequences of gliadin, especially 19-mer, bind to the highly expressed CXCR3 receptors, thus leading to increased intestinal epithelial permeability due to the increased release of zonulin. This increased permeability leads to the entrance of gliadin-derived toxic and immunogenic peptides into the lamina propria, which are deamidated by tissue transglutaminase (tTG2), resulting in a stronger binding affinity for HLA-DQ2 and DQ8 on antigen-presenting cells (APCs)^3^. In the following, innate and adaptive immunity mechanisms activate, causing chronic inflammation and tissue damage in the small intestine^4^. Innate and adaptive immune responses, especially the Th1 pathway, are critical pathogenic mechanisms in CeD. At the same time, the most important cytokines are IL-15, IL-27, IL-21, IL-2, IL-1β, IL-7, tumor necrosis factor α (TNF α) and interferon γ (IFN γ)^5,6^.The only acceptable treatment for CeD is strict and long-term adherence to a gluten-free diet (GFD). Despite following the GFD, 30–50% of patients experience recurrent symptoms and persistent lesions in the intestine. About 1% of patients do not respond to GFD (refractory CeD)^7^. Hence, there is a need for effective non-dietary treatment strategies to manage CeD. In this regard, enzyme therapy, which digests gliadin into non-immunogenic and non-toxic peptides and has anti-inflammatory properties, can be a promising therapeutic approach for celiac disease. Most gliadin-degrading enzymes target proline and glutamine residues, and by breaking gliadin into smaller peptides, they can reduce or eliminate the immune toxicity of gliadin and its derived toxic peptides^8^.

In our previous study, we showed that bromelain can degrade gliadin very well^9^. Bromelain is an effective proteolytic enzyme obtained from the fruit or stem of a pineapple (*Ananas cosmosus*). Bromelain has a broad specificity for protein breakdown, particularly in hydrophobic amino acid residues, and cleaves proteins in the carbonyl end of lysine, alanine, tyrosine, and glycine^10,11^. The molecular weight of bromelain is 23 to 37.0 kDa. Bromelain has an optimum temperature range of 37–60 °C and a pH range of 3–8^12^. Administration of bromelain Up to 750 mg/kg in dogs has been shown to have no toxic effects, and the lethal dose LD_50_ exceeds 10 g/kg in mice^13^. Bromelain possesses many therapeutic applications, including anti-inflammatory, anti-thrombotic, fibrinolytic, antimicrobial, and immunomodulatory properties^14^. Bromelain has been demonstrated to downregulate the expression levels and function of NF-κB and cyclooxygenase 2 (COX-2), significant inflammatory mediators that can convert arachidonic acid to the pro-inflammatory lipid prostaglandin E2. In addition, it can also modulate other inflammatory mediators such as IFNγ, IFNγ-mediated nitric oxide, TNF-α, IL-1 β, and IL-8. Therefore, bromelain is essential in a healthy immune system and inflammatory conditions by reducing cytokines and mediators^14,15^. Bromelain can be absorbed with significant bioavailability (up to 40%), lack of side effects, and without degradation or loss of proteolytic activity from the gastrointestinal tract, where most of the orally ingested enzyme is destroyed by gastric juices^16^. However, to improve the stability and function of bromelain, many studies have been conducted based on its Nano-formulation^17^.

Poly 3,4 Dihydroxy l phenylalanine (L-DOPA) is a dopamine derivative produced by self-polymerization of L-DOPA. Poly L-DOPA has more solubility than poly dopamine due to its additional carboxyl groups and greater negative charge. Due to the excellent adhesive properties of polydopamine, it has been extensively used as a coating agent for various applications, including functionalization, sensing, and drug delivery^18^. Polymerization of L-DOPA on the surface of silica nanoparticles leads to drug retention, prevention of degradation, and controlled release^19^.

In this study, to improve the stability and preservation of the enzymatic activity of bromelain, we loaded this enzyme into Poly L-DOPA nanocomposites coated on silica (Bromelain-Loaded Nanocomposites or BLNCs), and after determining the morphology, size, and zeta potential, the cytotoxicity of BLNCs was measured on the Caco-2 cell line. Also, the expression of CXCR3 and CCR5 genes in CaCo-2 cells treated with gliadin, gliadin-digested with BLNCs, and free bromelain were examined. Furthermore, after evaluating the release rate of bromelain from the nanocomposites, we evaluated the effect of untreated gliadin and gliadin treated with free bromelain and bromelain released from BLNCs on the innate immunity in PBMCs of healthy and celiac patients by measuring the level of pro-inflammatory cytokines (IL-1β, IL-6, and TNF-α). However, we also evaluated the expression level of IFN-γ, IL-10, and CTLA-4 in the PBMCs of these people, which also play a role in the regulation of acquired immunity.

## Results

### Size, morphology, and surface characterization of BLNCs

**Figure 1:**
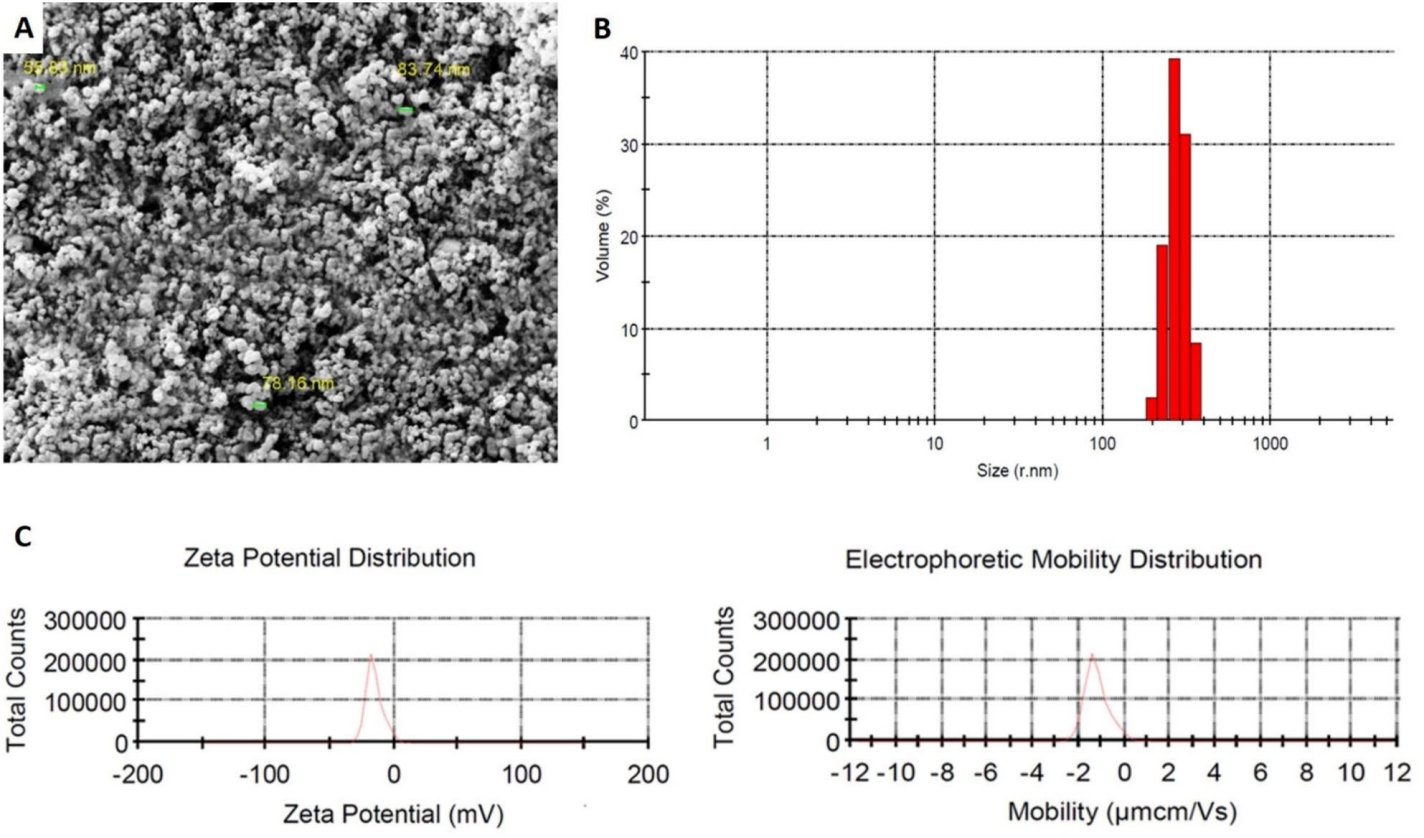
Characterization of BLNCs. (**A**) FE-SEM illustrations of BLNCs (20000X). Notably, nanocomposites accumulated and agglomerated when drying to prepare for FE-SEM analysis. Based on FE-SEM results, the size of the nanocomposites was measured around 56–84 nm. (**B**) The size distribution of BLNCs based on DLS analysis. (**C**) Curves of Zeta potential (ζ) and electrophoretic mobility distribution of the nanocomposites.

The FE-SEM (Field emission scanning electron microscopy) images illustrate that BLNCs were spherical. Nanocomposites had an average size of 72.57 nm (Fig. 1A). Also, the Dynamic Light Scattering (DLS) results indicated that 89.2% of BLNCs had a size between 229.3 and 307.6 nm (Fig. 1B). Poly-dispersity index (PDI) for these particles was 0.66, showing the nanocomposites had an acceptable size distribution. The Zeta potential (ζ) value of BLNCs and their electrophoretic mobility distribution were -15.2 mV and -1.2 μmcm/Vs, respectively (Fig. 1C).

### Investigating the chemical compositions of BLNCs

**Figure 2:**
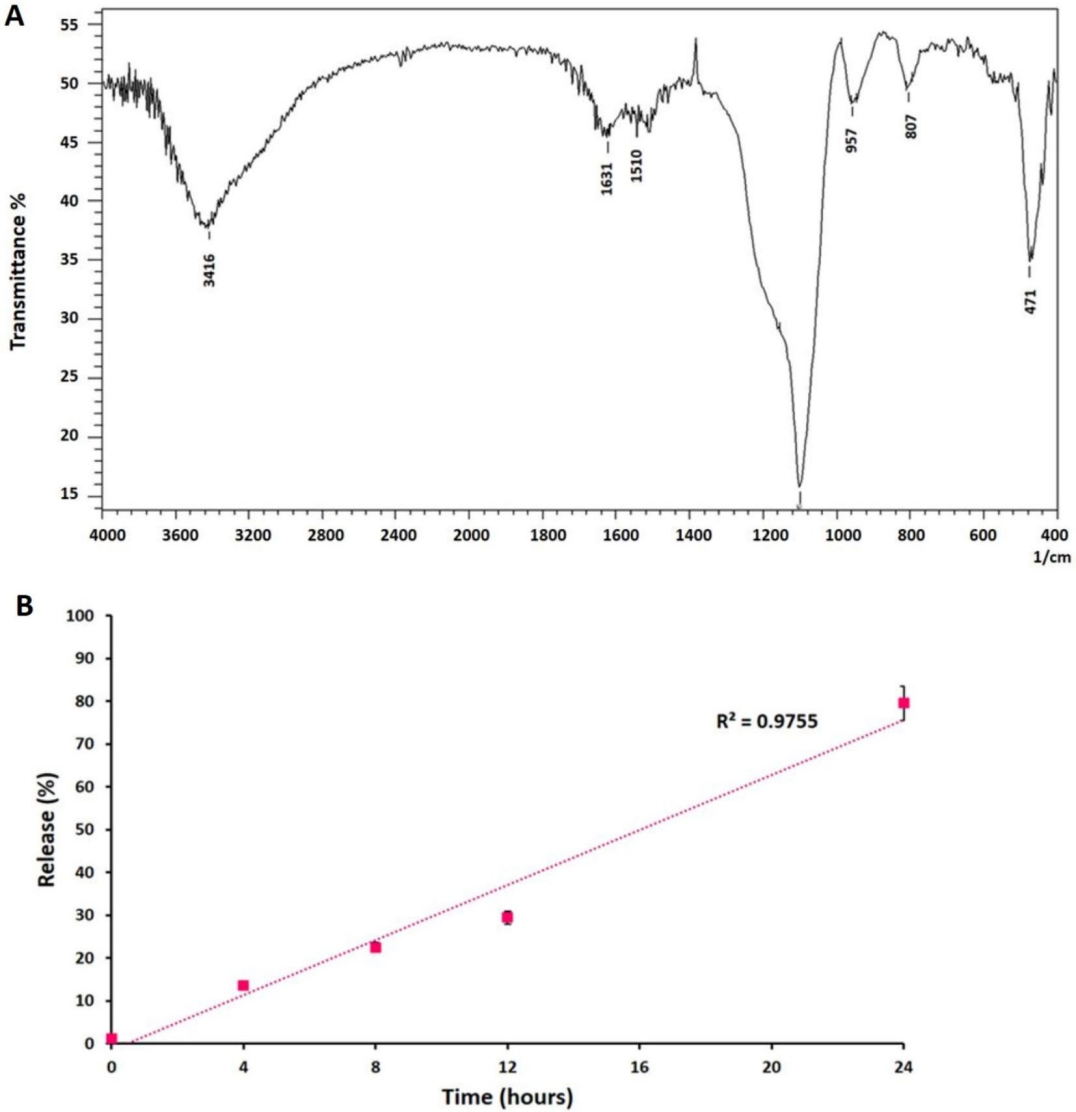
Chemical compositions and bromelain release rate of BLNCs. (**A**) FT-IR spectrum of the bromelain-loaded nanocomposite. (**B**) The release rate of bromelain from nanocomposite indicated that about 80% of bromelain was released after 24 h.

The FTIR (Fourier-transform infrared spectroscopy) spectra illustrated strong peaks at 1103 and 807 cm^−1^ related to the Si–O–Si stretching vibrations and a weak peak at 471 cm-1 related to its bending vibrations. Also, the absorbance of the –OH and –NH groups was observed at 3416 cm^−1^. The weak peaks at 1510 cm^-1^ and 1632 cm^−1^ were associated with the N–H bending vibration and the aromatic rings in the P-DOPA polymer, respectively (Fig. 2A).

### Loading capacity and release kinetics of bromelain

Results of loading capacity measurement showed that from 10 mg/mL of initial Bromelain, about 7.75 mg/mL of it was entrapped in Poly (L-DOPA) nanocomposites. According to the results, the entrapment efficiency of Bromelain in polymeric nanocomposite was 77.5%. Therefore, based on the weight of polymeric nanocomposites, the loading capacity of this enzyme was 77.5μg/mg. Also, the release rate of Bromelain from nanocomposites was almost constant, and 79.55% of this enzyme was released after 24 h (Fig. 2B). The release kinetics result showed that the nanocomposites slowly released Bromelain. So, polymeric nanocomposites positively affect the controlled release and protection of Bromelain.

### Degradation of gliadin by bromelain released from BLNCs

**Figure 3:**
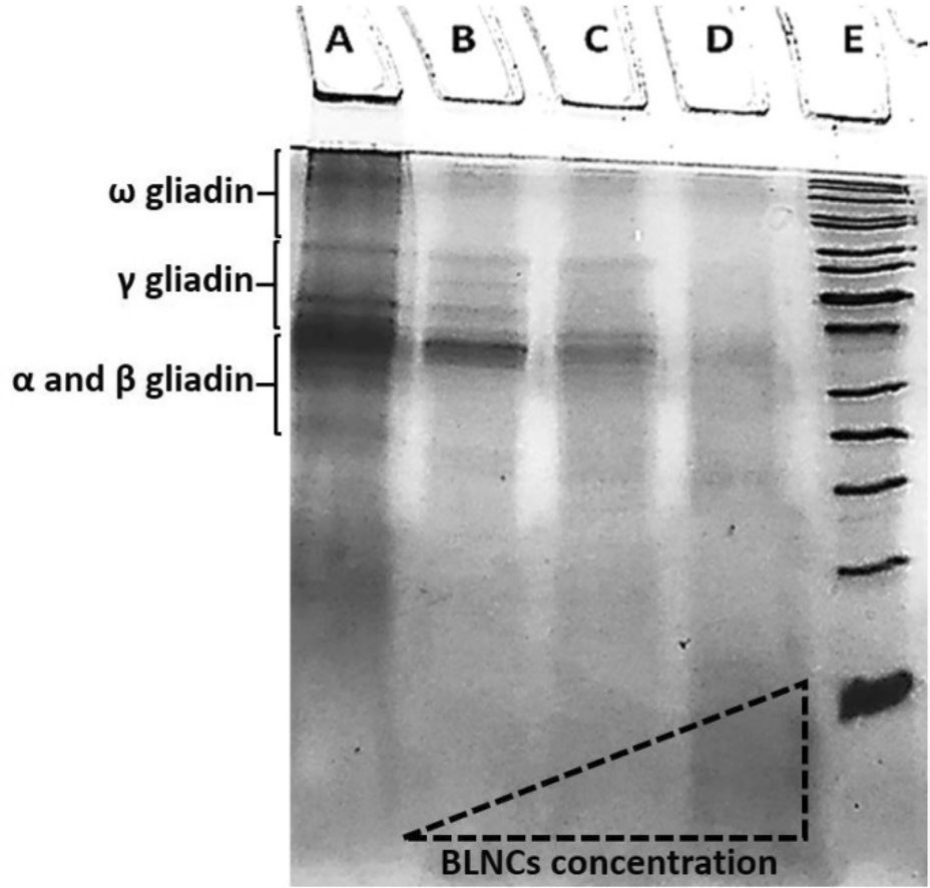
The SDS-PAGE gel of gliadin protein bands. Untreated gliadin (Column A) and gliadin after being treated by Bromelain that was released from different concentrations of BLNCs included 5mg/mL (Column B), 10mg/mL (Column C), and 20mg/mL (Column D). Column E is a protein-size marker. The original image of the gel (uncropped) is in the supplementary section (S1).

The pattern of protein bands in the SDS-PAGE gel shows that the Bromelain enzyme released from the BLNCs after 24 h has functioned and has been able to digest the gliadin protein. Also, as expected, with the increase in the concentration of BLNCs, the degradation rate of gliadin protein bands has intensified. So, with a 20 mg/ml concentration of BLNCs, almost all gliadin protein bands have been digested and disappeared compared to the control (Fig. 3). Since the loading efficiency of bromelain in nanoparticles was 77.5 μg/mg and on the other hand, after 24 h, almost 80% of this enzyme was released, it can be predicted that from 20 mg of BLNCs about 1550 μg of bromelain was released, that was able to digested gliadin bands with a concentration of 5 mg/ml.

### Cytotoxicity measurement by MTT assay

**Figure 4:**
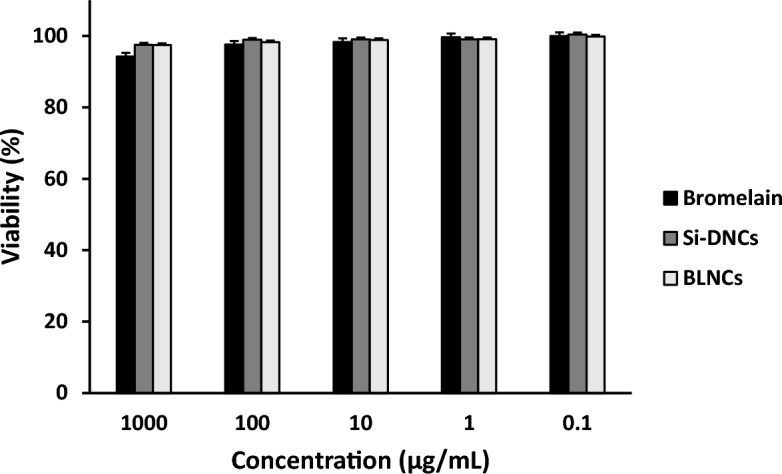
MTT assay for different concentrations of free Bromelain, BLNCs, and Si-DNCs against the Caco-2 cells after 24 h treatment. Error bars represent the standard deviation of three independent replicates for each concentration.

To determine the cytotoxicity of Bromelain alone, Silica-poly(L-DOPA) nanocomposites (Si-DNCs), and bromelain-loaded nanocomposites (BLNCs) against the Caco-2 cells, a standard MTT assay was performed. The results showed that Bromelain alone has no significant inhibitory effects on cell growth. Also, Si-DNCs are safe for these cells and have no significant impact on their growth inhibition at all concentrations. Regarding the toxicity of BLNCs, there was no significant difference between the group of Bromelain alone and Si-DNCs (*p* > 0.05), which shows that the loading of Bromelain on Si-DNCs did not cause toxicity against cells. Although, with the loading of Bromelain in nanocomposites, the toxicity of the enzyme decreased slightly in 24 h compared to Bromelain alone (Fig. 4). Therefore, it can be concluded that Si-DNCs are safe carriers for bromelain delivery.

### Gene expression of CCR5 and CXCR3 in Caco-2 cells

**Figure 5:**
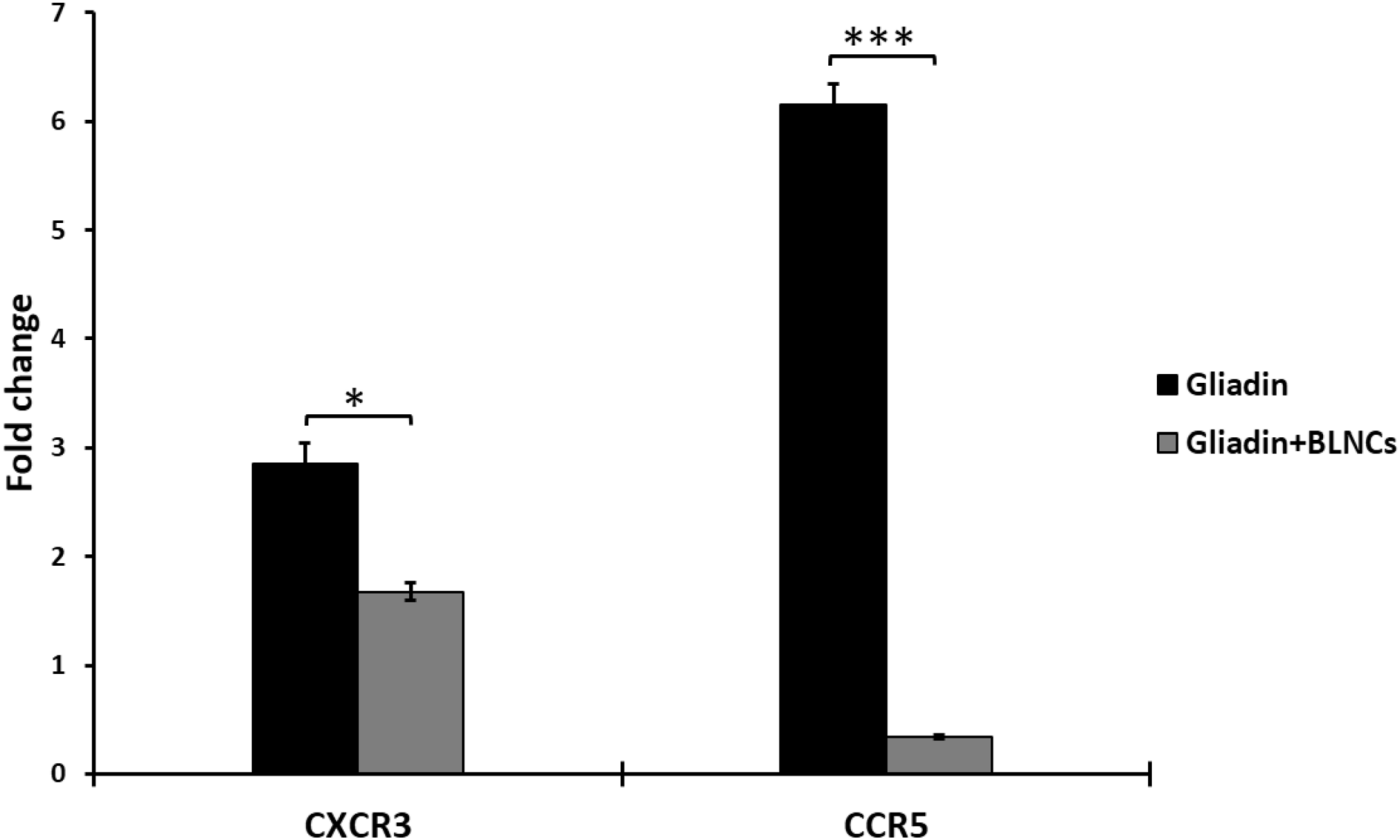
Graph of the relative expression level of genes CXCR3 and CCR5 in Caco-2 cells treated with gliadin and gliadin digested with BLNCs. In this figure, * means *p*-Value ≤ 0.05, *** means *p*-Value ≤ 0.001. Student's t-test was used as a statistical test. *p*-values ≤ 0.05 were considered statistically significant. Error bars represent the standard deviation of three independent replicates for each gene.

Today, the importance of the expression of genes CCR5 and CXCR3 in the pathogenesis of celiac disease is well known. The results show that treatment of Caco-2 cells with intact gliadin increases the expression of CXCR3 and CCR5 genes. As it is known, compared to untreated cells, the expression of genes CXCR3 and CCR5 in Caco-2 cells treated with gliadin increased 2.84 and 6.14 times, respectively. When the Caco-2 cells were treated with digested gliadin that was treated with enzymes released from nanoparticles for 24 h, the expression of genes CXCR3 and CCR5 decreased significantly (*p* < 0.05), so that the expression of genes CXCR3 and CCR5 decreased to 1.67 and 0.34 times, respectively (Fig. 5).

### Cytokine measurement by enzyme-linked immunosorbent assay (ELISA)

**Figure 6:**
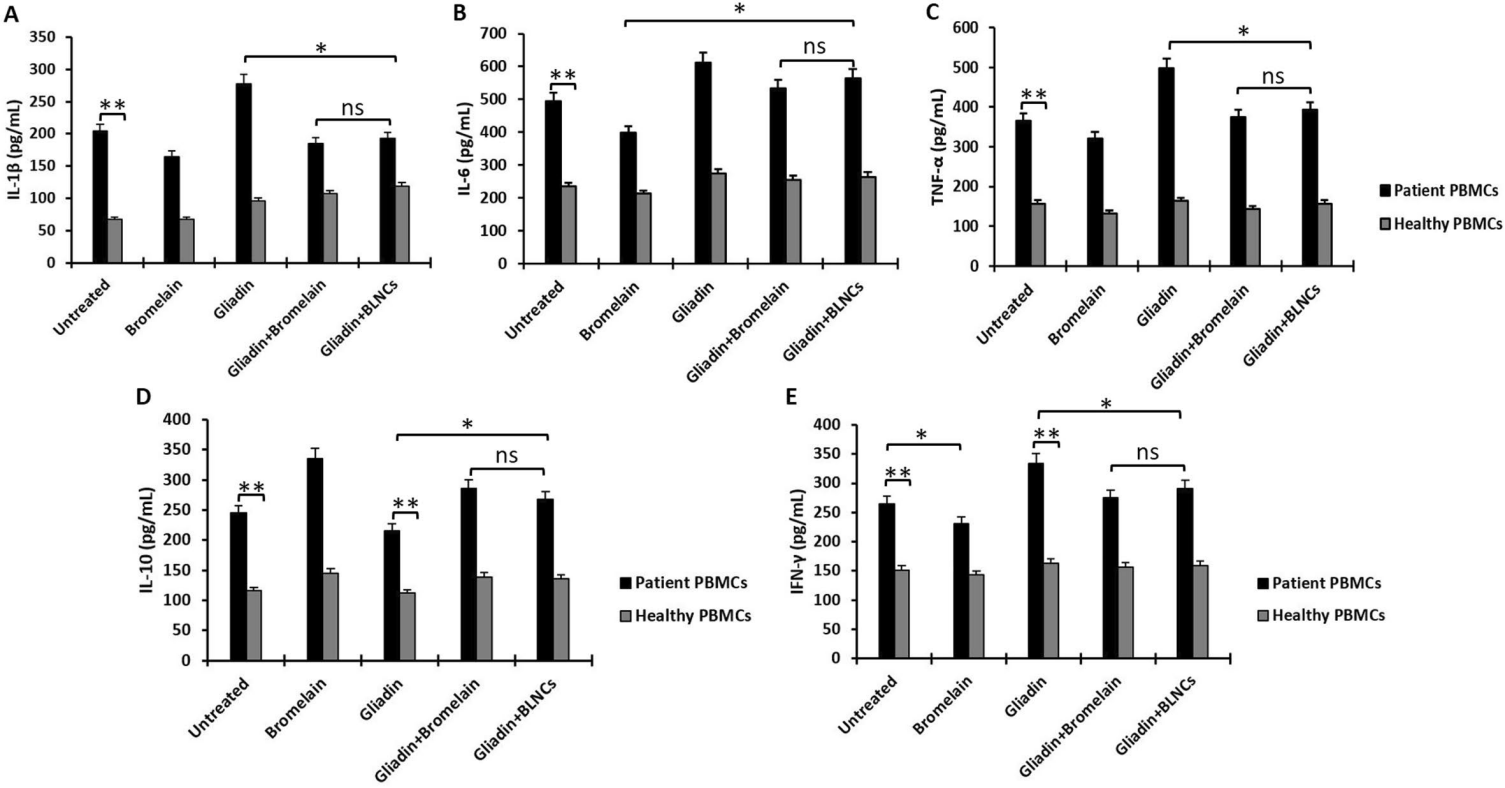
This illustration of the ELISA test shows the production of (**A**) IL-1β, (**B**) IL-6, (**C**) TNF-α, (**D**) IL-10, and (**E**) IFN-γ cytokines by the PBMCs of celiac disease patients and healthy people. The investigated groups include untreated cells treated with intact gliadin; gliadin digested with bromelain, gliadin digested with BLNCs, and bromelain alone. To perform the ELISA test in each of the treated and non-treated groups, the PBMCs of 7 people with celiac disease and 7 healthy people were used. The mean cytokines level obtained from the PBMCs of all healthy and celiac disease people was statistically analyzed. Error bars represent the standard deviation. One-way ANOVA was used as a statistical test. P-values ≤ 0.05 were considered statistically significant. In this figure, ns means non-significant, * means * p* value ≤ 0.05, and ** means *p* value ≤ 0.01.

To evaluate the production of inflammatory cytokines (IL-1β, IL-6, TNF-α, and IFN-γ) and IL-10 in PBMCs of patients and healthy persons treated with gliadin and gliadin digested by BLNCs, an ELISA was conducted. Evaluation of cytokine levels in supernatant obtained from the PBMCs culture of healthy and patient persons showed that the level of IL-1β, IL-6, TNF-α, and IFN-γ in untreated patients' PBMCs was much significantly higher than in untreated healthy PBMCs (*p* < 0.005). Treatment of patients with PBMCs with intact gliadin caused a significant increase in the secretion of IL-1β, IL-6, IFN-γ, and TNF-α cytokines in their culture medium compared to untreated cells (*p* < 0.05). However, this treatment decreased IL-10 production compared to the untreated group (*p* > 0.05). On the contrary, celiac PBMCs treated with Bromelain reduced the production of pro-inflammatory cytokine compared to untreated celiac PBMCs (*p* < 0.05), indicating the anti-inflammatory role of Bromelain. This treatment significantly increased the level of IL-10 production compared to the untreated state of the same cells (*p* < 0.05). Nevertheless, when patient PBMCs were treated with gliadin digested by free Bromelain, and BLNCs, they showed a significant decrease in the production of IL-1β, IL-6, IFN-γ, and TNF-α cytokines compared to the patient PBMCs treated with intact gliadin but production of IL-10 under the effect of this treatment in patient PBMCs showed a significant increase compared to when the same cells were treated with intact gliadin (Fig. 6).

### CTLA-4 expression in PBMCs of celiac disease patients and healthy people

**Figure 7:**
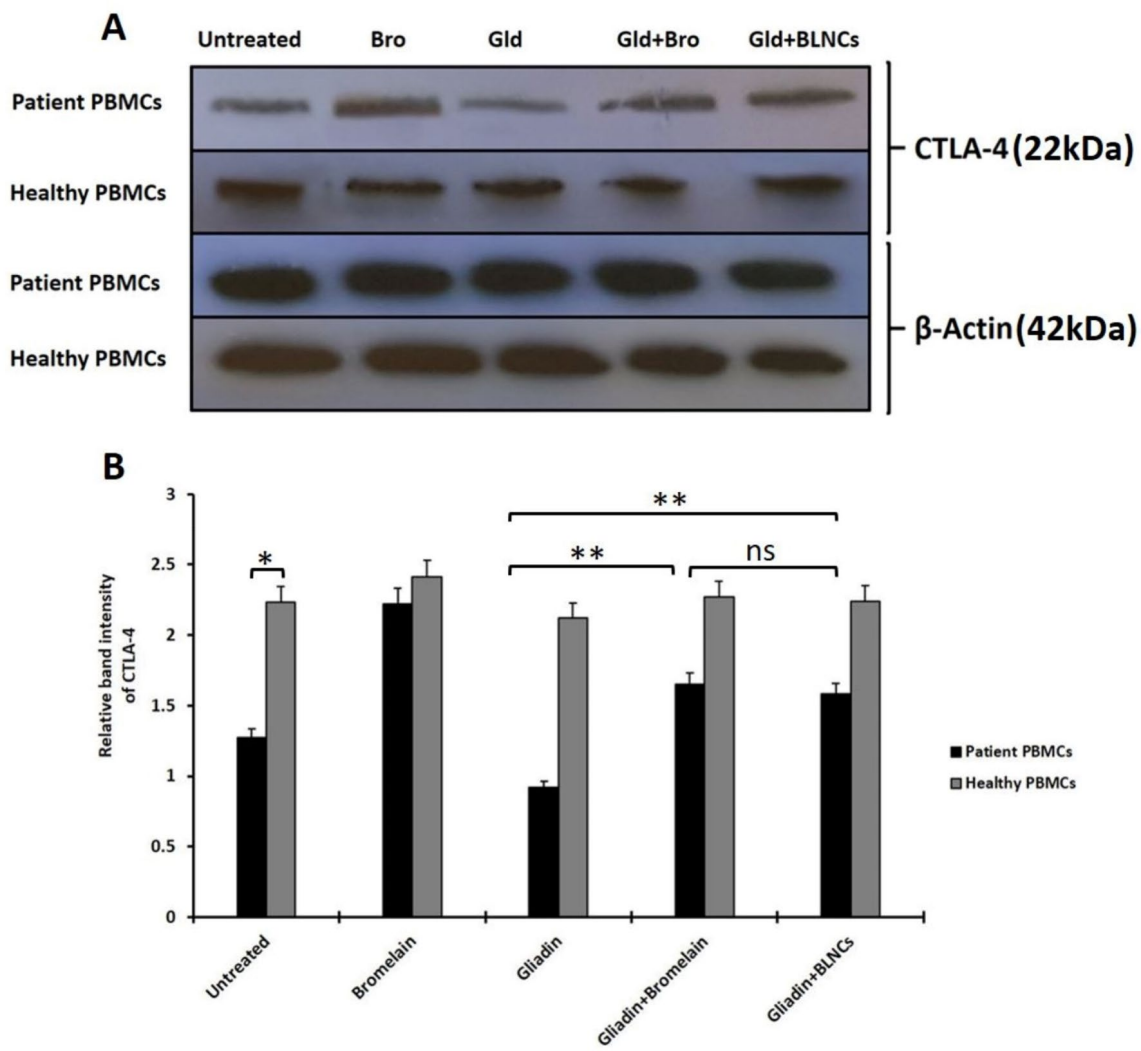
Western blot and its relative band intensity. (**A**) A representative image of protein bands of Western blotting was performed to detect the protein levels of CTLA in the treated and untreated PBMCs of celiac disease patients and healthy people and (**B**) the relative intensity of CTLA-4 protein expression in the mentioned treatments. To perform the western blot in each of the treated and non-treated groups, the PBMCs of 7 people with celiac disease and 7 healthy people were used. The mean band intensity obtained from each group was statistically analyzed. Error bars represent the standard deviation. One-way ANOVA was used as a statistical test. *p* values ≤ 0.05 were considered statistically significant. In this figure, ns means non-significant, * means *p* value ≤ 0.05, ** means *p*-value ≤ 0.01. The original image of the blotting film (uncropped) is in the supplementary section (S2).

To evaluate the protein expression of CTLA-4 in patients and healthy PBMCs treated with gliadin and gliadin digested by BLNCs, a western blot was conducted (Fig. 7A). The density of CTLA-4 bands was determined to present the relative intensity of protein expression (Fig. 7 B). β-actin protein was used as internal control and to normalize the expression of CTLA-4 in test groups. Analysis of protein bands showed that the expression level of CTLA-4 in untreated patients' PBMCs was significantly lower than in untreated healthy PBMCs (*p* < 0.005). Bromelain alone considerably increased the amount of CTLA-4 expression in the PBMCs of celiac patients compared to the same cells without bromelain treatment (*p* < 0.05). However, bromelain did not significantly affect the expression of CTLA-4 in the PBMCs of healthy people. When the PBMCs of celiac patients were treated with intact gliadin, the decrease in CTLA-4 expression was significantly apparent compared to the untreated group (*p* < 0.05). However, this treatment did not affect protein expression in healthy PBMCs, and they were almost the same in both treated and untreated groups (*p* > 0.05). Then, treatment of the patients' PBMCs with gliadin with bromelain encapsulated in nanoparticles (Gliadin + BLNCs) showed a significant increase in protein expression compared to the group treated with intact gliadin (*p* < 0.05). The treatment of healthy PBMCs with gliadin digested with bromelain (Gliadin + BLNCs) showed no significant difference in terms of CTLA-4 expression compared to the group treated with intact gliadin (*p* > 0.05). Compared to gliadin digested with BLNCs, gliadin decomposed with free bromelain increased the CTLA-4 expression in the cells of celiac patients. However, this change in expression was not significantly different in these two types of treatment (*p* > 0.05).

## Discussion

Even though celiac disease has been known for a long time, there is still no definite way to prevent and treat this disease^20,21^. However, the best therapeutic and preventive strategy for clinical manifestations of celiac disease is to follow a gluten-free diet, which, besides being very expensive, is not available to everyone. Also, using gluten in non-food items such as medicines and supplements is sometimes unavoidable. Therefore, due to the incomplete response of many patients to the GFD, as well as the significant problems that exist with adherence to the GFD, the development of new non-dietary treatment methods to manage symptoms of this disease to eliminate gliadin toxicity, reducing the inflammation and repair tissue damages seems necessary. Today, gliadin-degrading enzymes of various origins have received much attention as a promising strategy for treating celiac disease. Enzymes derived from various sources that can break down the immunogenic peptides of gluten are commonly known as glutenases^22^. These enzymes can cleave the glutamine-proline bonds in the protein sequence. Therefore, glutanases can be suitable candidates for proteolytic digestion of gluten. Endopeptidase-40 (E40) and latiglutenase (ALV003) are the most important glutanases that have been used to digest gluten. E40 is a secreted protein obtained from the soil bacterium Actinoallomurus A8, and its active form is produced by *Streptomyces lividans TK24* through recombinant technology^22^. Latiglutenase is a combination of two glutenases, EP-B2 (From barley seed endosperm) and SC-PEP (A proline-specific endoprotease from *Sphingomonas capsulata*)^23^. Also, E40 has been previously demonstrated to break down the highly immunogenic 33-mer peptide and the entire gliadin proteins, leading to a significant reduction in the release of interferon (IFN)-γ when exposed to T cells isolated from individuals with CeD^24^. However, to date, no protease has been introduced that specifically and completely reduces the content of immunogenic gliadin in gastric conditions below the toxicity threshold for celiac patients^25^. Papain-like cysteine proteases are promising candidates as gluten and gliadin-degrading enzymes. These enzymes are very stable and are often found in proteolytic environments such as apoplast, vacuole, and lysosomes of plant cells^26^. Papain, chymopapain, caricaine, bromelain, and ficin from this family have substrate specificity and strong proteolytic activity^27^. In our recent study, the digestive effects of a bromelain and ficin mixture on gliadin were first predicted by computational methods based on their cleavage sites. Next, their toxicity, allergenicity, and binding tendency to HLA-DQ2 and HLA-DQ8 molecules were simulated with 19-mer toxic peptides and intact gliadin. In the following, it was proved by methods such as SDS-PAGE, HPLC, and CD that these two enzymes are capable of breaking down gliadin in vitro and in simulated stomach environments. Finally, the effects of gliadin and the toxic peptide derived from it before and after treatment with a mixture of Bromelain and Ficin were determined on cell toxicity against Caco-2 cells and the expression of genes involved in tight junctions in these cells^9^. The proteolytic effects on gliadin, antioxidant activity, wound healing, and anti-inflammatory and anti-ulcer properties of bromelain were the most important reasons for choosing this enzyme in the present study. Bromelain was loaded in different nanopolymers to increase its stability and activity protection. For the first time, we encapsulated this enzyme in poly(L-DOPA) nanoparticles. So, the results of measuring its size, morphology, PDI, and entrapment efficiency are unique for these nanoparticles and may not be comparable with similar studies with other polymers. However, bromelain has been used to investigate the antimicrobial activity and anticancer properties of nanoparticles with different types of polymers. The results of our study showed that the average size of BLNCs based on the FE-SEM was 72.57 nm, which showed some differences compared to some studies. For example, in one study, the average size of poly (lactide-co-glycolide) acid or PLGA based on TEM was 20 to 35 nm^28^. In another study, bromelain was loaded in hyaluronic acid (HA) grafted PLGA copolymer whose average size was 146.3 nm based on DLS and 30–40 nm based on TEM. SEM results for these nanoparticles showed that HA-PLGA nanoparticles have a spherical shape with smooth surfaces^29^. Also, BLNCs synthesized in our study are almost spherical, and the amount of PDI based on the DLS for them was determined to be 0.66, while the PDI of Bromelain loaded in PLGA and HA-PLGA nanoparticles was 0.095 and 0.340, respectively^28,29^. These differences in nanoparticles could be due to the different types of polymer used in these nanoparticles.

Also, the entrapment efficiency and loading capacity for BLNCs were 77.5% and 77.5 μg/mg, respectively. In other studies, the entrapment efficiency and loading capacity for bromelain were different for other polymers. Bromelain was encapsulated in a poly (acrylic acid) (PAA) polymer in one study. Conjugation of bromelain on these nanoparticles reduced the enzyme activity of bromelain to 63%. The loading capacity in this study was reported as 253 μg/mg^30^. Although the bromelain binding capacity was lower in our nanoparticles (77.5 μg/mg), the activity of the enzyme released from the BLNCs was almost equal to that of free bromelain, which did not show any decrease in activity. The results of the bromelain release rate from BLNCs showed that using poly (L-DOPA) nanocomposites can cause the slow and constant release of this enzyme from BLNCs. We did not measure the release of bromelain for more than 24 h, but in the first 12 h, 29.5% of bromelain was released from the BLNCs, which indicates an appropriate release. After 24 h, 79.5% of bromelain was released from nanoparticles. The bromelain released from the BLNCs was able to digest gliadin well. Biocompatibility and lack of cytotoxicity of polymers are some of the most essential principles for applying Nano-carriers for drug delivery. Sometimes, the combination of drug and polymer can show toxicity. For this purpose, we investigated the toxicity of free bromelain, Silica-poly(L-DOPA) nanocomposites, and BLNCs on CaCo-2 cells by the MTT method. The results showed that BLNCs do not have any toxicity on these cells, and the percentage of cell survival after 24 h is 97.46% at the highest concentration of BLNCs (1000 mg/mL). In a study, bromelain was loaded into katira gum polymer, and their toxicity on human skin fibroblast cells showed more than 95% survival, which confirmed the results of our study^31^. In CeD, the chemokine receptor CCR5 (CD195) is likely involved in gluten-specific CD4 + T cell migration into the gut lesion^32^. The expression of significant levels of CCR5 mRNA in each of the epithelial cell lines of the small intestine and the inducibility of its gene expression under the influence of various factors such as stromal cell-derived factor 1alpha and macrophage inflammatory protein (MIP)- 1alpha or MIP-1beta have been proven^33^. Also, many studies have shown CXCR3 expression in human intestinal enterocytes^34^. In this study, we treated CaCo-2 cells with gliadin and gliadin treated with bromelain and evaluated the expression of genes CCR5 and CXCR3 under these conditions. In a study, the treatment of CD4 + T cells in celiac disease cells with gluten increased the gene expression of the CCR5, which indicates the worsening of the disease with the increase of this receptor^32^. We showed that using bromelain released from BLNCs with digestive effects on gliadin and its immunomodulatory effects causes a decrease in CCR5 expression compared to the group treated with intact gliadin. Therefore, it can be assumed from this preliminary study that bromelain can improve the symptoms of celiac disease. Several studies have shown that blocking CCR5 and CXCR3 with monoclonal antibodies and fusion proteins plays a role in inhibiting or attenuating autoimmune diseases^35^. However, in an animal study, the use of a CXCR3 antagonist was able to moderate psoriasis, although it was ineffective in clinical trials^36^. Also, using CCR5 antagonists in rheumatoid arthritis (RA) did not have much therapeutic effect on this disease^37^. Today, many studies have proven the anti-inflammatory properties of bromelain, but limited studies have suggested its use to improve celiac symptoms^38,39^. However, in some studies such as the study of Ivan Cruz-Chamorro et al., the effects of wheat gluten on the level of pro-inflammatory cytokines in the PBMCs of healthy adults have been mentioned^40^. In our study, the effects of pure gliadin on the level of pro-inflammatory cytokines and the expression level of CTLA-4 in people with celiac disease and healthy people were evaluated. Our study on the PBMCs of celiac patients showed that intact gliadin can increase the production of pro-inflammatory cytokines (IL-1β, IL-6, TNF-α, and IFN-γ) and decrease the production of anti-inflammatory cytokines (IL-10) which confirms the results of other studies on the anti-inflammatory properties of bromelain. For the first time, we have shown that when gliadin is broken down with free bromelain and then PBMCs are added, the production of pro-inflammatory cytokines decreases significantly, but IL-10 secretion significantly increases. Also, treating these cells with BLNCs had very similar effects to the groups treated with gliadin digested by free bromelain, which shows that bromelain encapsulated in BLNCs has maintained its function. When we treated the PBMCs of celiac patients and healthy people with bromelain alone without gliadin, it was found that in addition to digesting gliadin, bromelain can exert anti-inflammatory effects by applying its inherent anti-inflammatory properties.

These results show that digesting gliadin and the anti-inflammatory effects of bromelain can modulate the production of inflammatory cytokines and induce the release of IL-10. Cytotoxic T lymphocyte antigen 4 (CTLA-4) is necessary for regulating and modulating T lymphocyte-mediated inflammatory responses. A common CTLA-4 haplotype shows a strong association with coeliac disease^41^. Also, CTLA-4 haploinsufficiency has been reported in coeliac patients^42^. Therefore, the importance of CTLA-4 in the control of inflammatory responses in celiac disease is proven. So far, CTLA-4 protein expression in PBMCs of celiac patients treated with bromelain, intact gliadin, and gliadin treated with bromelain has not been investigated. For the first time, western blot results in our study showed that the PBMCs of celiac patients treated with intact gliadin significantly reduced the expression of CTLA-4. However, when gliadin is digested with a free bromelain enzyme, the expression of CTLA-4 increases in PBMCs. This increase in CTLA-4 expression can be attributed to a decrease in the toxic and inflammatory effects of the gliadin after degradation with bromelain and the inherent anti-inflammatory effects of bromelain. The comparison of CTLA-4 expression due to the digestion of free bromelain and bromelain encapsulated in BLNCs on gliadin confirms the preservation of the function of bromelain released from BLNCs.

## Conclusion

The ability to digest gliadin and toxic peptides derived from it by bromelain and its anti-inflammatory effects can suggest a promising strategy to treat or improve symptoms caused by celiac disease. One of the most important limitations of using enzymes is their instability in physiological conditions. Encapsulation of enzymes in nano-polymers can overcome this limitation. In this study, we encapsulated bromelain enzyme in Poly (L-DOPA) nanocomposite for the first time. The entrapment efficiency of bromelain in these nanoparticles was appropriate, and 79% of it was released after 24 h. These nanoparticles had no toxic effects on the cells, and more than 97% of the cells remained alive at the highest concentration of BLNCs. Also, the efficiency of bromelain released from nanoparticles was proven by different methods. For the first time, the effects of gliadin, gliadin decomposed with bromelain, and bromelain enclosed in dopamine nanoparticles were evaluated on PBMCs affected by celiac disease. It was found that bromelain has decomposition effects. Bromelain and its inherent anti-inflammatory effects could reduce the production of IL-1, IL-6, TNF-α, and IFN-γ and, on the other hand, cause an increase in IL-10 and CTLA-4 in the blood of these patients. Considering these therapeutic potentials of bromelain-loaded nanocomposites, further evaluation of in vivo studies and clinical trials is suggested.

## Methods and materials

### Chemicals, reagents, media, and cell line

Gliadin from wheat (G3375), and Bromelain from pineapple stem (B4882), were purchased from Sigma-Aldrich Co (St. Louis, MO, USA). Acetic acid, Bradford solution (ultra-pure water, coomassie brilliant blue g-250, ethanol 95%, orthophosphoric Acid 85%), tris (hydroxymethyl)-aminomethane (tris base), tris (hydroxymethyl)-aminomethane hydrochloride (tris–HCl) and glycine, were purchased from Merck Millipore (Darmstadt, Germany). Acrylamide (99.9%0, Bis (N, N'-Methylene-bis-acrylamide), sodium dodecyl sulfate (SDS), ammonium persulfate (APS), and triton X-100 were purchased from Bio-Rad Co. N, N, N′, N′-Tetramethylethylenediamine (TEMED) was purchased from Sigma-Aldrich Co (St. Louis, MO, USA). Bromophenol blue was purchased from Plus-One Co. Milli-Q ultra-pure (UPW) water was also used. A human colon cancer cell line (Caco-2 with NCBI code C139 from the National Cell Bank, Pasture Institute of Iran) was used for the cytotoxicity test. RPMI (Roswell Park Memorial Institute medium) 1640 medium and Fetal Bovine serum (FBS) were bought from Gibco (Carlsbad, CA, USA). Penicillin and streptomycin, trypan blue dye, trypsin, MTT dye [3-(4, 5-dimethylthiazol-2-yl)-2, 5-diphenyltetrazolium bromide], glutaraldehyde, and dimethyl sulfoxide (DMSO) were purchased from Sigma-Aldrich (St. Louis, MO, USA). Total RNA Purification Kit and cDNA synthesis kit were purchased from Jena Bioscience, (Jena, Germany). For Real-time PCR, Real Q Plus 2 × Master Mix Green made by the AMPLIQON (Odense, Denmark) was used.

### Synthesis of the bromelain-loaded nanocomposites (BLNCs)

To coat the silica nanoparticles with Poly L-DOPA molecularly imprinted polymers, 0.05 g of silica nanoparticles were mixed in 15 mL tris buffer (10 mM, pH 8.5). Then 0.05 g of L-DOPA was added to the mixture and shaken on a stirrer at room temperature for 1 h. Then, 1 mL of Bromelain enzyme with a concentration of 10 mg/mL in deionized water was added to the mixture and stirred for 12 h at room temperature. The resulting mixture was centrifuged to remove the free enzyme and extra monomeric L-DOPA and washed three times with deionized water. To understand the loading capacity of bromelain in poly (L-DOPA) nanocomposites, the concentration of free bromelain in this supernatant was measured by a standard Bradford method, and according to the initial concentration of bromelain, its loading capacity in the nanocomposite was obtained. Entrapment efficiency and loading capacity of nanocomposites were determined using the following formulas:

$$\begin{gathered} {\text{Entrapment efficiency }}\left( \% \right){ } = { }\frac{{\left( {{\text{Initial Conc}}^{*}{\text{ of bromelain}}} \right){ }{-}\left( {\text{Conc* of bromelain in Supernatant}} \right){ }}}{{\text{Initial Conc*of bromelain}}} \times 100 \hfill \\ {\text{Conc}}^{*} \, = {\text{Concentration }}\left( {{\text{mg}}/{\text{mL}}} \right) \hfill \\ {\text{Loading capacity}} = \;{\text{total}} \hfill \\ \end{gathered}$$

Finally, the silica nanoparticles coated with molecularly imprinted polymers were dispersed in 20 mL of deionized water and stored in the refrigerator^43^.

### BLNCs characterization

To investigate the surface characteristics, morphology, and size of BLNC, the FE-SEM method by a ZEISS Gemini SEM 360 (Jena, Germany) was used according to the standard protocol.

The particle size distribution and Zeta potential (ζ) of bromelain-loaded nanocomposite (BLNC) were determined using Zetasizer (Malvern Instruments Ltd, Malvern, United Kingdom) based on Dynamic Light Scattering (DLS). BLNCs with a refractive index of 1.59 dispersed in water with a refractive index of 1.33 entered the Zetasizer. The particle size distribution was measured at 25 °C for 60 s and with a counting speed of about 400,000 counts per second (kcps).

The chemical composition of synthesized BLNCs was investigated using Fourier transform infrared (FTIR) spectroscopy (6700 Thermo Nicolet) at 400 cm^−1^ to 4000 cm^−1^^44^.

### Measurement of bromelain release kinetics from BLNCs

To investigate the release rate of bromelain, BLNCs were dissolved in PBS (pH = 7.2) at 37 °C and 150 shakes per minute. Briefly, the supernatant solution was sampled at intervals of 0, 2, 4, 6, 8, 10, 12, and 24 h. Next, the concentration of bromelain was measured based on a standard Bradford assay. In each interval, the bromelain concentration was determined by comparing them with the BSA (Bovine Serum Albumin) standard curve, and the release kinetics graph was drawn.

### Validation of bromelain activity after releasing from bromelain-loaded nanocomposite

Gliadin was prepared at 5mg/mL in ethanol 70%. Then, the prepared gliadin was treated with bromelain released from three different concentrations of BLNCs (5, 10, and 20 mg/mL) after 24 h of release in V/V% 1:1 (Gliadin: BLNCs) at 37 °C for 24 h. Samples were stored at − 20 °C until further chemical analysis. A 12% SDS-PAGE was used to investigate the digestion of gliadin by BLNCs. 20 μL of samples and five μL of prestained protein marker (BLUelf, GeneDirex) were loaded in this gel, which was run under 100 V for 120 min. The running buffer was Tris–glycine (pH = 8.3). Next, the gel was fixed with a fixation solution for 60 min and then washed three times with Ultra-Pure Water. After staining the gel with colloidal coomassie brilliant blue (G-250) staining solution overnight, it was de-stained with 1% v/v acetic acid solution for 1 hour^45^.

### Subjects of ex vivo study

To obtain the PBMCs, a total of seven celiac patients and seven healthy persons (without specific infection, other autoimmune diseases, allergies, and immunodeficiency) aged 15–40 years in Semnan University of Medical Sciences were recruited. All cases were monitored by a gastroenterology specialist during the study. Patients with celiac disease were selected based on serological and pathological indications under the supervision of a gastroenterologist. All patients followed a gluten-free diet. Healthy subjects were selected in terms of age and gender similar to the patient group. Acute clinical indications in selected patients in this study were controlled by following a gluten-free diet for at least 6 months. This study was approved by the Medical Ethics Committee of Semnan University of Medical Sciences (Semnan, Iran) with the ethics code of IR.SEMUMS.REC.1399.017. We confirm that all experiments were performed under relevant guidelines and regulations. Also, we confirm that informed consent was obtained from all subjects or their legal guardian(s) (In the case of minors).

### Sample collection, PBMC culture, and their treatment

In this study, blood samples were taken from each volunteer (7 celiac patients and seven healthy persons). Each sampling process took 10–15 mL of blood from each person. In this project, the expression levels of IL-1β, IL-6, TNF-α, IL-10, and IFN-γ cytokines and CTLA-4 protein are determined in peripheral blood mononuclear cells (PBMCs) after treatment with undigested gliadin and bromelain- digested gliadin. PBMCs were obtained from blood samples by Ficoll-Hypaque (Ficoll™ Paque Plus; cat. no. 17–1440-03; GE Healthcare) density gradient centrifugation as previously described^46^. Collected PBMCs were cultured in RPMI media with 10% FBS and 1% Pen/Strep antibiotics in standard conditions (5% CO_2_ and 37℃) based on a standard protocol previously described^46^. After the cells reached a suitable growth, they were treated with gliadin, Bromelain-digested gliadin with BLNC, and Bromelain alone (500μg/mL) for 48 h. Also, one group was considered a negative control and not treated with any combination. Then, the cells of each treated group and negative control were scraped and collected by centrifugation. The supernatant obtained from these cell sediments was kept at − 20 °C for cytokine analysis, and their cell pellet was held at -80 °C for the western blot test.

### Cytotoxicity assay of bromelain-loaded nanocomposite against Caco-2 cells

To determine the toxicity of BLNCs, free Bromelain and Silica-poly(L-DOPA) nanocomposites (Si-DNCs), an MTT assay against the Caco2 cell line was conducted according to current standard protocol^47^. Five concentrations (1000, 100, 10, 1, and 0.1 µg/mL) were selected for BLNCs, free Bromelain, and Si-DNCs. Finally, the absorbance was measured at 545 nm using a microplate reader (STAT FAX 2100, BioTek, Winooski, USA). The cell's viability percentage and half-maximal inhibitory concentration (IC50) were calculated as follows:

$${\text{Toxicity}}\% = \left( {1 - \frac{{{\text{mean OD of sample}}}}{{{\text{mean OD of control}}}}} \right) \times 100$$

$${\text{Viability\% = 100 - Toxicity\% }}$$

### Real-time PCR

The effect of gliadin and gliadin treated by BLNCs on CXCR3 and CCR5 mRNA expression was investigated by Real-time PCR (qRT-PCR) based on the SYBR Green method. Primer sequences for CXCR3 and CCR5 were designed using AllelID software and evaluated by oligo analyzer and primer blast tools. The primers were provided by SinaClon (SinaClon, Tehran, Iran) and are listed in Table 1. GAPDH (Glyceraldehyde-3-Phosphate Dehydrogenase) gene, a housekeeping gene, is used as an internal control. Briefly, Caco2 cells were cultured in a 12-well microplate at a density of 1 × 10^5^ cells/well for 24 h at optimal conditions. Next, growth media was removed, and the cells were treated with the fresh RPMI medium containing 10% FBS and 500 µg/mL of samples (Gliadin before and after treatment with BLNCs) and incubated for 72 h under optimal conditions. Then, the total RNA was extracted using the blood/cultured cell Total RNA Purification Kit (Jena Bioscience, Jena, Germany) according to the manufacturer’s protocol. The quality and quantity of total RNA were detected using a NanoDrop® spectrophotometer (Hitachi, Tokyo, Japan). In the following, the cDNA was synthesized by a SCRIPT cDNA Synthesis Kit (Jena Bioscience, Jena, Germany). Then cDNA, primers, and Real Q plus 2 × Master Mix Green with high ROX (Ampliqon, Denmark) was added to Real-Time PCR strips, and qPCR was performed using an ABI system (Applied Biosystems; Thermo Fisher Scientific, Inc.). qPCR was conducted as follows: Initial denaturation at 95 °C for 15 min; 40 cycles of denaturation (at 95 °C for 20 s) and annealing (at 52–62 for 30 s); and extension at 72  °C for 30 s. 2^-∆∆Ct^ method was used to interpret the results^48^.

**Table 1:** Sequence of primers.

Primer name	Sequence
CXCR3	Forward: 5′-ATCAACTTCTACGCAGGA-3′ Reverse: 5′-GGTGGCATGAACTATGTT-3′
CCR5	Forward: 5′-GGCTGTGAGGCTTATCTT-3′ Reverse: 5′-TTCAGGAGAAGGACAATGTT-3′
GAPDH^19^	Forward: 5′-GTCTCCTCTGACTTCAACAGCG-3′ Reverse 5′-ACCACCCTGTTGCTGTAGCCAA-3′

### Measurement of inflammatory and anti-inflammatory cytokines by ELISA

To determine the effects of gliadin, gliadin-digested with BLNCs, gliadin-digested with bromelain, and free bromelain on the production of IL-1β (Cat number: DY201, R&D Systems, United States), TNF-α (Cat number: DY210, R&D Systems, United States), IL-6 (Cat number: D6050, R&D Systems, United States), IFN-γ (Cat number: ab174443, Abcam, MA, USA), and IL-10 (Cat number: ab185986, Abcam, MA, USA) by PBMCs of healthy and patients people, a ELISA for supernatant of PBMCs culture obtained from section "Cytokine measurement by enzyme-linked immunosorbent assay (ELISA)" was performed.

In brief, known concentrations of each standard cytokine and the experimental samples were added and incubated in polystyrene microtiter plates coated with an antibody against the appointed cytokine and after treatment by blocking buffer (5% w/v non-fat dry milk in PBS buffer), followed by incubation with an enzyme-linked polyclonal antibody directed to the cytokine. Next, a substrate solution (3,3',5,5'-tetramethylbenzidine) for the enzyme was added, and the color development was stopped by adding 2N (2 Normal) H_2_SO4. The absorbance was measured with a microtiter plate spectrophotometer at 450 nm. The amount of each cytokine in each sample was determined from a standard curve generated in each assay and expressed as picograms per milliliter (pg/mL). The reproducibility of all measurements was within 10% in our laboratory^49^.

### Western blot analysis

A western blot was performed to investigate the CTLA-4 expression in PBMC treated with gliadin and gliadin digested by BLNCs. For this purpose, Collected PBMCs in “Cytokine measurement by enzyme-linked immunosorbent assay (ELISA)” Section  (patients and healthy PBMC treated with gliadin, gliadin digested by BLNCs, free bromelain, gliadin digested by free bromelain, and an untreated well was also considered as control) were lysed with RIPA buffer and the lysates were removed by centrifugation at 14,000 rpm for 20 min at 4 °C. The BCA Protein Quantification kit was used according to the manufacturer's instructions to determine protein concentration. The cell lysates were mixed with an equal volume of 2X Laemmli sample buffer. Next, 20 μg of lysates were subjected to SDS-PAGE and transferred to a 0.2 μm Immune-Blot™ polyvinylidene difluoride (PVDF) membrane (Cat No: 162-017,777; Bio-Rad Laboratories, CA, USA). The following, membranes were blocked with 5% BSA (Cat No: A-7888; Sigma Aldrich, MO, USA) in 0.1% Tween 20 for 1 h. The membranes were incubated with anti-CTLA-4 (Cat No: ab231949, Abcam) and anti-β actin loading control antibodies (Cat No: ab8227, Abcam) for 1 h at room temperature. After washing thrice with TBST, the membranes were incubated with goat anti-rabbit IgG H&L (HRP) (Cat No: ab6721; Abcam) secondary antibody. Then, the membranes were incubated with enhanced chemiluminescence (ECL) for 2 min, and protein expression was normalized to β-actin. The gel analyzer Version 2010a software (NIH, USA) was used for the densitometry of protein bands. Finally, the percentage area under the curve of each band was divided by the percentage area under the curve of its corresponding β-actin band, and calculated values were compared between groups as described previously^48^.

### Statistical analysis

GraphPad Prism software (version 9.0.0 (121)) was used for statistical analysis and determination of the mean, variance, and standard deviation of the data obtained from the experiments. *p*-values < 0.05 were considered statistically significant.

### Supplementary Information

Supplementary Information.

## Data Availability

Data is contained within the article or supplementary material.
