# Supplementary material for: Bromelain-loaded nanocomposites decrease inflammatory and cytotoxicity effects of gliadin on Caco-2 cells and peripheral blood mononuclear cells of celiac patients
Source: Sci Rep. 2023 Dec 1;13:21180. doi: 10.1038/s41598-023-48460-3 (PMC10692183; doi:10.1038/s41598-023-48460-3)
Supplement: Supplementary file 1 — Supplementary Information. [file 41598_2023_48460_MOESM1_ESM.docx]

**S1. Original SDS-PAGE gel image (Figure 3 in text)**


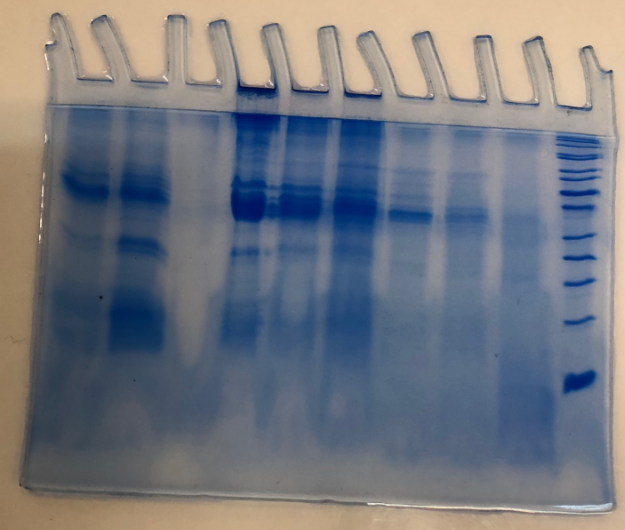


**S2. Original western blot**


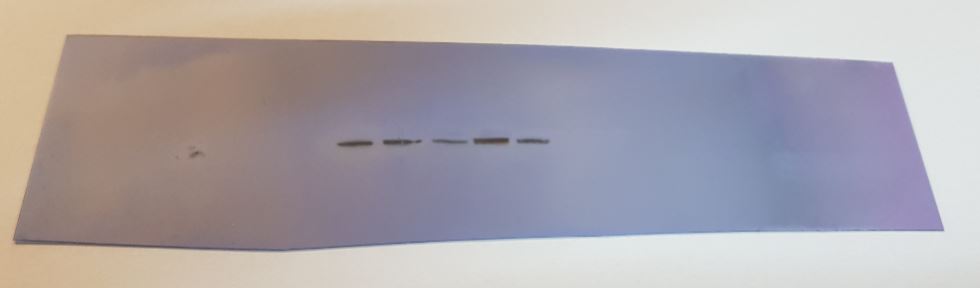


**Patient PBMCs- CTLA4**


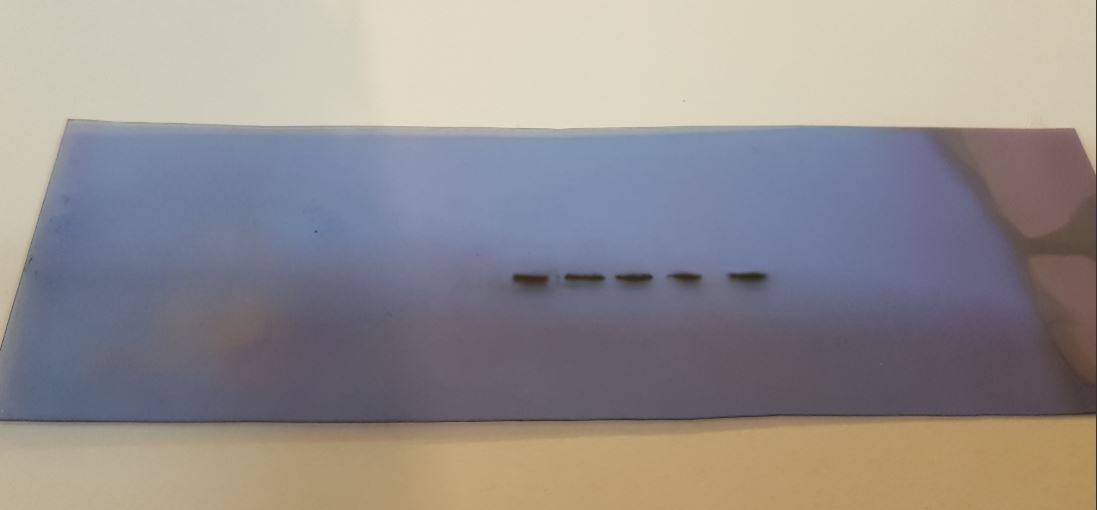


**Health PBMC- CTLA4**


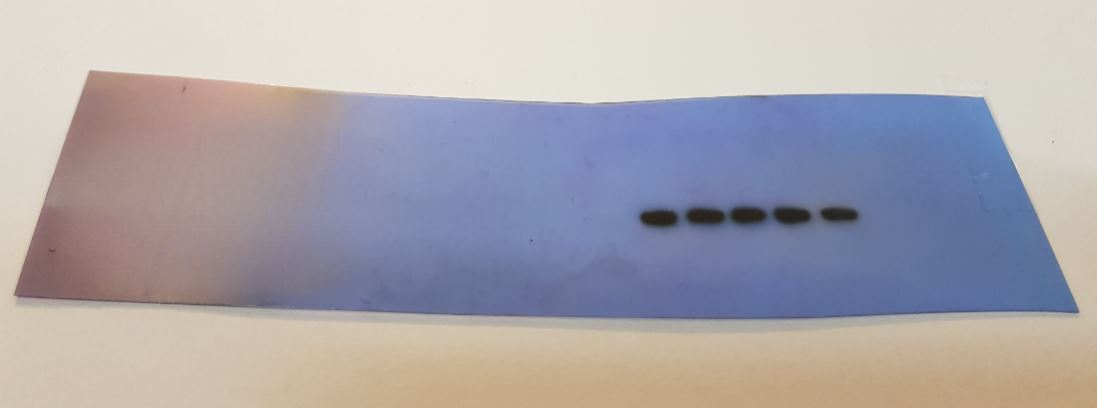


**Patient PBMCs- β-Actin**


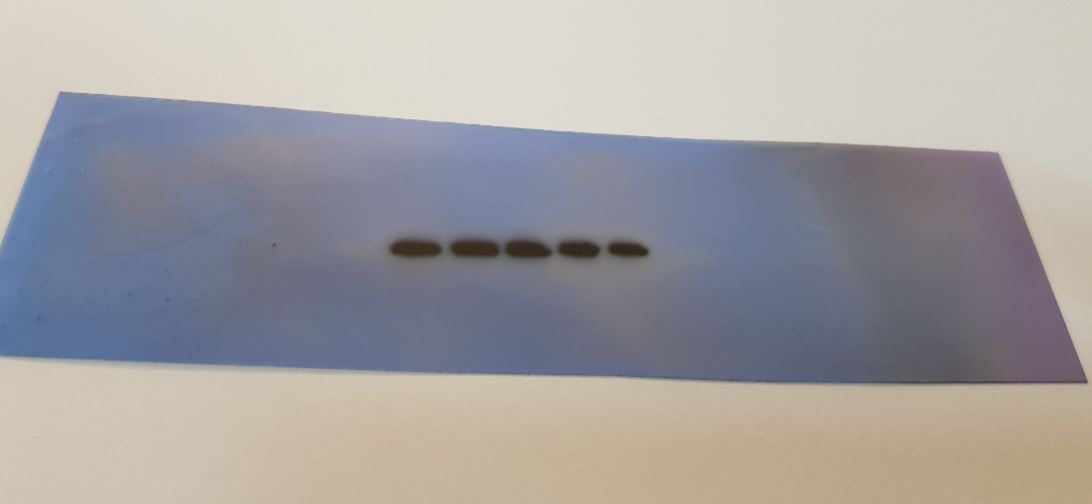


**Health PBMCs- β-Actin**
